# The Repair Strategy for Event Coverage Holes Based on Mobile Robots in Wireless Sensor and Robot Networks †

**DOI:** 10.3390/s19225045

**Published:** 2019-11-19

**Authors:** Yaoming Zhuang, Chengdong Wu, Hao Wu, Hao Chu, Yuan Gao, Li Li

**Affiliations:** 1 Faculty of Robot Science and Engineering, Northeastern University, Shenyang 110819, China; 2 Engineering Faculty, University of Sydney, Sydney, NSW 2006, Austria; 3 College of Information Science and Engineering, Northeastern University, Shenyang 110819, China; gaoyuan@stumail.neu.edu.cn; 4 JangHo School of Architecture, Northeastern University, Shenyang 110819, China

**Keywords:** mobile robots, event coverage holes, multi-constrained circumstances, path plan, wireless sensor and robot networks

## Abstract

In the application of the wireless sensor and robot networks (WSRNs), there is an urgent need to accommodate flexible surveillance tasks in intricate surveillance scenarios. On the condition of flexible surveillance missions and demands, event coverage holes occur in the networks. The conventional network repair methods based on the geometric graph theory such as Voronoi diagram method are unable to meet the conditions of flexible surveillance tasks and severe multi-restraint scenarios. Mobile robots show obvious advantages in terms of adaptation capacity and mobility in hazardous and severe scenarios. First, we propose an event coverage hole healing model for multi-constrained scenarios. Then, we propose a joint event coverage hole repair algorithm (JECHR) on the basis of global repair and local repair to apply mobile robots to heal event coverage holes in WSRNs. Different from conventional healing methods, the proposed algorithm can heal event coverage holes efficaciously which are resulted from changing surveillance demands and scenarios. The JECHR algorithm can provide an optimal repair method, which is able to adapt different kinds of severe multi-constrained circumstances. Finally, a large number of repair simulation experiments verify the performance of the JECHR algorithm which can be adapted to a variety of intricate surveillance tasks and application scenarios.

## 1. Introduction

The research of event coverage hole repair is significant in wireless sensor and robot networks (WSRNs) [1,2,3,4]. The conventional repair methods based on the geometric graph theory such as Voronoi diagram method pay attention to the coverage hole repair problem in an one-fold unconstrained scenario [5]. Nevertheless, in a real environment, it is indispensable to use different kinds of sensors to heal event coverage holes on the basis of flexible surveillance demands [6]. Meanwhile, the repair process is limited by different kinds of intricate restraints. In the method of healing the coverage holes, the conventional healing ways redeployed by humans and airplanes which throw many nodes randomly [7,8]. WSRNs are frequently applied in the hazardous and severe scenarios. Redeployment by humans will waste lots of effort and money, which is hazardous for humans [9]. Although the nodes are scattered at random by airplanes to heal the coverage holes, the healing effectiveness cannot be ensured [10], and the existing coverage holes cannot be healed. Moreover, unnecessary nodes will cost many network resources. With the continuous improvement in robot technology, mobile robots are widely used. Mobile robots are able to serve in the hazardous and severe scenarios and are fast moving to an accurate place [11]. Therefore, we propose to apply mobile robots to accurately heal event coverage holes in WSRNs.

Different from conventional coverage holes that resulted in sensor invalidation, the event coverage holes usually resulted in the flexible surveillance missions. For example, a large conference center will hold different competitions, conferences, or concerts. Different kinds and quantities of nodes are required to be redeployed in diverse places of the stadium according to diverse events. While the surveillance event changes, lots of coverage holes in the surveillance scenarios appear. Conventional deployment approaches are deterministic deployments. The conventional strategy is merely able to be used to an onefold scene [12]. If the surveillance events and demands vary, or the nodes break in the surveillance scenarios, it will cost lots of effort and funds to redeploy and heal the networks. The conventional strategy is unable to satisfy the realtime demands of the scenarios [13,14].

Repair conventional coverage holes only need one single kind of nodes. In order to adapt to different surveillance scenarios and flexible surveillance demands, single kind of nodes is unable to heal networks effectively and meet a variety of intricate surveillance demands.

Event coverage holes usually appear in hazardous and severe scenarios. When healing the networks, it is restricted by kinds of severe restraints. Even if the surveillance scenario is consistent, the network healing is restricted by lots of restraints according to various surveillance events [15]. For example, in wild hill surveillance, there are different surveillance demands for the hill at different periods. During the war, the hill can be monitored for war. In peacetime, hill fire surveillance and poaching surveillance are conducted in the hill. With different surveillance tasks, under the condition of limited sensor resources, the healing restraints of networks are also different. Under the task of war surveillance, it is necessary to heal the network quickly and accurately. Therefore, the healing of networks is restrained by severe time restraints and error restraints; in hill fire surveillance, it is necessary to control the repair cost and to minimize the energy consumption of the networks. Therefore, the healing procedure is limited by strict cost restraints and energy restraints; in poaching surveillance, it is necessary to quickly identify poachers, accurately confirm the location of poachers, and control the repair cost of the event coverage holes. Therefore, the healing of networks is restrained by severe time restraints, error restraints, and cost restraints.

On the basis of these analyses, it is necessary to propose the reasonable healing strategies for flexible surveillance demands. Our innovations are as follows:

- It is the first time that the healing issues of event coverage holes are put forward to flexible surveillance demands. Diverse kinds of nodes are used to heal event coverage holes in WSRNs effectively.
- For the first time, the multi-restraint surveillance scenarios are proposed to solve network healing problems. Focusing on the intricate multi-restraint scenarios and restricted resources, we propose an event coverage hole healing model with various restraints.
- For the first time, the mobile robots are used to heal coverage holes in WSRNs. The mobile robots are applied to take different kinds of nodes to heal networks with a reasonable healing strategy. On the basis of global repair and local repair, a joint event coverage hole repair algorithm (JECHR) is proposed to distribute sensor resources and plan the optimal repair route in WSRNs.

The structure of the paper is organized as follows. Related works are elaborated in Section 2. The healing model is presented aiming at multiple restraints in Section 3. The local event coverage hole repair algorithm is presented in Section 4. The global repair algorithm is presented in Section 5. The joint repair algorithm is presented for WSRNs with multiple restraints in Section 6. In Section 7, the network healing simulation experiments and performance comparisons are carried out. At last, the conclusion is elaborated in Section 8.

## 2. Related Works

In the study of conventional event coverage hole healing, it is common to use one single kind of sensors to heal the networks. Faced with various surveillance demands, one-fold kind of nodes cannot satisfy the surveillance demands in the networks. Sun presents a healing algorithm based on event-driven policy to accomplish maximum event coverage region [16]. On the basis of the above event-driven scheme, in [17], Sun presents an event-driven coverage control protocol (ECCP) to enhance the event monitoring region. The above research is restricted to the application of one-fold kind of nodes to heal networks. One-fold kind of nodes cannot satisfy various surveillance demands. In [18], Alam presents an event coverage strategy to regulate the surveillance radius in real time. The coverage strategy overcomes over-provisioning in the networks, extends lifetime and extendibility, and improves surveillance capability in a cost-efficient method. The above coverage strategy is only able to improve the event coverage performance provisionally that is not able to heal all the event coverage holes in WSRNs.

The network healing is limited by various restraints in intricate scenarios [19,20,21]. In [22], Gao first proposes the energy restraints in event coverage. Jiang presents a distributed and energy-saving event k-coverage algorithm (DEEKA) aiming at intricate scenarios in the underwater sensor networks. DEEKA algorithm first takes into account the impact of severe underwater scenarios on data acquisition and transmission [23]. In practice scenarios, the networks are limited by various severe restraints. In [24], Hasan takes into account the data transmission problem in multi-restraint scenarios.

Mobile robots are able to accomodate to hazardous and severe scenarios. In [25], Soares first presents to apply mobile robots to acquire information in WSRNs. In [26], Guo presents a high performance distributive two-hop coloring algorithm to set up collision-free communication connections in WSRNs. In [27], the mobile robots are used to help relocate the location of the nodes. On the basis of the above methods, in [28], Arezoumand uses spanning tree as a detection method to assist robots in deploying the nodes.

In the research of WSRNs, planning path of multi-robots efficiently in terms of various surveillance tasks is an urgent need to be addressed. Yuan first presents optimum robot path planning issue in WSRNs [29]. In [30], Lee presents a distance-aware robot routing (DAR) method in WSRNs to select the optimal routing for mobile robots by taking into account the features which are distinguished from the packet routing. In [31], Imeson presents a new language in which uncontinuous route planning issues for mobile robots are addressed. On the basis of the above method, in [32], Imeson proposes a new method that simplifies the SAT-TSP language to the general TSP language in senior robotic route planning issues. In [33], Trigui takes into account the issue of dispatching object positions visited by mobile robots. In order to solve the above problem, a fuzzy logic algorithm is proposed to settle the multi-target TSP aiming at multiple robot system. In [34], a bi-objective ant colony optimization (ACO) algorithm on the basis of memetic method is presented to settle the multi-robot patrolling issues.

The research on event coverage hole healing, multi-restraint network scenario, WSRNs and multi-robot system path planning are elaborated. On the basis of above analyses, mobile robots can effectively adapt to intricate scenario in WSRNs. The current research is mainly aimed at the path planning of multi-robot systems. It is not considered how to apply mobile robots to heal event coverage holes in complex application scenarios. So, we put forward an event coverage hole healing model and joint repair algorithm on the basis of global repair and local repair to heal event coverage holes in multi-restraint WSRNs.

## 3. The Event Coverage Hole Healing Model

### 3.1. Main Idea

Unlike the conventional coverage hole healing issue, the event surveillance is mainly used in hazardous and severe scenarios. It is usually limited by diverse restraints when healing the event coverage holes. The multi-restraint network healing problem is highly intricate. Therefore, the multi-restraint network healing issue is translated into an unrestricted multi-objective healing issue. By transformation, the unrestricted multi-objective healing issue is able to be settled by effective multi-objective optimization algorithm. The above method is able to boost the efficiency of the solutions and simplify the complexity of the issue.

### 3.2. Problem Formulation

Without loss of generality, the healing issue under multi-restraint is stated as follows:

(1) $$\begin{array}{lll} \max & f(\alpha )=f(\alpha_{1},\alpha_{2},\ldots ,\alpha_{n}) & \\ s.t. & g_{i}(\alpha )=g_{i}(\alpha_{1},\alpha_{2},\ldots ,\alpha_{n})\leq 0 & \;(i=1,2,\ldots ,p)\\ & h_{i}(\alpha )=h_{i}(\alpha_{1},\alpha_{2},\ldots ,\alpha_{n})=0 & \;(i=p+1,\ldots ,q) \end{array}$$

f(α) is defined as the coverage objective function. *i* denotes the quantity of restraint functions. $\alpha =(\alpha_{1},\alpha_{2},\ldots ,\alpha_{n})\in T\subset R^{n}$ denotes diverse kinds of nodes where $\alpha =(\alpha_{1},\alpha_{2},\ldots ,\alpha_{n})\in T\subset R^{n}$ denotes an *n*-dimensional decision variable. $T=\{\alpha_{t}\in R^{n}|d_{t}\leq \alpha_{t}\leq u_{t},t=1,2,\ldots ,n\}$ denotes the object space. $d_{t}\in R$ and $u_{t}\in R$ are the lower bound and upper bound of $\alpha_{t}$. $g_{i}(\alpha )$ represents inequality restraint function. Similarly, $h_{i}(\alpha )$ represents equation restraint function. 

The feasible region of the event coverage hole healing issue with equation restraints is smaller in comparison to the object space. The equation restraints can be translated into inequality restraints.

(2) $$\left|h_{i}(\alpha )\right|-\lambda \leq 0\;\;(i=p+1,\ldots ,q)$$

λ denotes the tolerability for translating the equation restraints into inequality restraints. λ represents a small positive constant.

When figuring out the optimization issue with restraints, it is essential to balance the relation between the objective function and the restraint violation degree. The restraint violation degree is deemed as another objective function. The multi-restraint healing issue is translated into an unrestricted multi-objective issue.

(3) F(α)=(f(α),V(α))

In Equation (3), F(α) denotes the multi-objective function by transformation. f(α) represents the objective function of the multi-restraint healing issue before transformation. V(α) is the restraint violation degree, namely, the amount of the restraint violation values. It can be seen from the healing model, that the multi-restraint healing issue is translated into an unrestricted bi-objective issue.

## 4. Local Event Coverage Hole Repair Algorithm

### 4.1. Main Idea

The invasive weed optimization algorithm (IWO) is a random search algorithm that evolved from the principle of weed evolution in nature. The IWO algorithm imitates the basic process of weed invasion, proliferation, growth, reproduction, and competitive extinction, with strong robustness and adaptability. The main feature of the IWO algorithm is that the weeds will gradually grow in specific areas. In the end, the weeds will dominate, which is similar to the evolutionary process. The IWO algorithm usually consists of four steps:

1. Population initialization: A specific number of weeds are randomly and evenly distributed in the n-dimensional search space.
2. Population reproduction: Each individual in the IWO algorithm will generate offspring in its own specific area. Among them, the number of offspring generated by each individual is determined by the confidence of individuals. The number of offspring will vary linearly with the confidence of individuals.
3. Offspring spatial distribution: The offspring generated by individuals are randomly distributed around the parent in a normal distribution to further enhance the regional search capability.
4. Individual selection: As the number of individuals in the population increases, the maximum value of the population is finally reached. Therefore, it is necessary to propose a reasonable selection mechanism to ensure the diversity of the population and to make the population evolve in a better direction.

In the application of the invasive weed optimization algorithm, the offsprings generated by each individual are dispersed around parents. If the offsprings are dispersed near by parents, this allocation is able to accomplish favourable network healing effect. On the basis of the above analysis, we present a local repair algorithm on the basis of IWO algorithm.

### 4.2. The Distribution Strategy on the Basis of Confidence

The IWO algorithm is adopted as the local repair strategy in the joint repair algorithm. In the IWO algorithm, the quantity of offspring reflects the capability of parents. The conventional restricted optimization algorithms are only inclined to feasible solutions. Lots of feasible solutions with poor objective function value are reserved. Meanwhile, lots of infeasible solutions with small restraint violation degree are ignored. On one hand, this leads to the feasible solutions with inferior objective function values that generate many offspring. On the other hand, the excellent infeasible solutions that can provide guidance for optimizing direction are fewer. In order to settle the above issues, a distribution strategy based on confidence is presented to optimize the quantity of offspring. The distribution strategy trades off the objective function and the restraint violation degree via confidence. The confidence is confirmed via the proportion of feasible solutions. The confidence distribution strategy is denoted as:

(4) $$Confid(\alpha )={\left[\gamma f_{norm}{\left(\alpha_{k}\right)}^{2}+(1-\gamma )V_{norm}{\left(\alpha_{k}\right)}^{2}\right]}^{1/2}$$

(5) $$\left\{ \begin{array}{l} f_{norm}(\alpha_{k})=\frac{f(\alpha_{k})-f{\left(\alpha \right)}_{\min}}{f{\left(\alpha \right)}_{\max}-f{\left(\alpha \right)}_{\min}}\\ V_{norm}(\alpha_{k})=\frac{V(\alpha_{k})-V{\left(\alpha \right)}_{\min}}{V{\left(\alpha \right)}_{\max}-V{\left(\alpha \right)}_{\min}} \end{array}\right.$$

In Equations (4) and (5), γ represents the proportion of feasible solutions. $f_{norm}(\alpha_{k})$ and $V_{norm}(\alpha_{k})$ denote the normalized values of the objective function value $f(\alpha_{k})$ and the restraint violation degree $V(\alpha_{k})$, separately. The number of offspring is:

(6) $$\left\{ \begin{array}{l} Num(Weed)=\left\lfloor W_{\max}-f_{k}(W_{\max}-W_{\min})\right\rfloor \\ f_{k}=\frac{Confid(\alpha_{k})-Confid{\left(\alpha \right)}_{\min}}{Confid{\left(\alpha \right)}_{\max}-Confid{\left(\alpha \right)}_{\min}} \end{array}\right.$$

In Equation (6), $W_{\max}$ is the maximum number of offspring, and $W_{\min}$ is the minimum number of offspring. ⌊⌋ represents rounding down.

### 4.3. The Polynomial-Based Distribution Function

During the process of local repair, finding methods to regulate the step factor efficiently is a pressing need to be addressed. As a result, we present a polynomial-based distribution function as the distribution operator. The distribution operator is applied to generate diverse distribution values in multiple dimensionalities in the decision space. Then these distribution values are applied to generate offspring.

The distribution function on the basis of polynomial adopts the present value as the mean. The variance is confirmed by the step length *s* of the IWO algorithm. The step length *s* determines the degree to which the distribution vector deviates from the current vector. The polynomial-based distribution function is denoted as:

(7) $$PD(\varepsilon )=\frac{1}{2}(s+1){\left(1-\left|\varepsilon \right|\right)}^{s},\varepsilon \in (-1,1)$$

(8) $$\varepsilon =\left\{ \begin{array}{l} \sqrt[{s+1}]{2\eta }-1,\;\;\eta <0.5\\ 1-\sqrt[{s+1}]{2(1-\eta )},\eta \geq 0.5 \end{array}\right.$$

In the Equations (7) and (8), ε represents the adjustment factor, ε∈(−1,1). η denotes a random number, η∈(0,1). The final distribution vector is denoted as:

(9) $$d=b+\varepsilon \varphi_{\max}$$

In Equation (9), d represents the distribution vector. $\varphi_{\max}$ denotes the maximum adjustment of the deviation vector b.

In conclusion, if the step length *s* is large enough, the distribution vector is approaching to the parent vector. So, applying the distribution function on the basis of polynomial as the distribution operator is able to accomplish local repair effect in WSRNs.

### 4.4. The Selection Strategy on the Basis of Non-Dominated Sorting

When the population reaches the allowable maximum quantity, the individual with poor objective function value will be removed. So as to select individuals more effectively during the local repair process, the selection strategy on the basis of non-dominated sorting is used to rank individuals. The selection strategy on the basis of non-dominated sorting is able to get rid of individuals with inferior objective function value. Among the non-dominated individuals, each individual is distributed to the non-dominated front of the different layers. The selection strategy of the IWO algorithm is defined as:

**Definition 1.**  *If the individuals are on different Pareto fronts, the individual at the higher Pareto front is selected*.

**Definition 2.**  *If the individuals are on the same Pareto front, the individual with lower restraint violation degree is selected*.

By ranking, individuals with superior objective function value and low restraint violation degree are retained and conduct to the next iteration. The local repair algorithm is denoted in Algorithm 1.

**Algorithm 1** The Local Event Coverage Hole Repair Algorithm Based on IWO Algorithm.1: Begin2:  Input: $\alpha =(\alpha_{1},\alpha_{2},\ldots ,\alpha_{n})\in T\subset R^{n}$;3:  W=PD (α);4:  $W_{m}=PM\;(W)$;5:  $\alpha^{\prime }=\alpha \cup W_{m}$;6:   *If*
$\alpha^{\prime }=\alpha \cup W_{m}$ exceeds the allowable maximum amount of the population, then $\alpha =SE(\alpha^{\prime })$;7:   End *if*.8: End

In Algorithm 1, W denotes the offspring; $W_{m}$ represents the mutation offspring; $\alpha^{\prime }$ denotes the merged new population; PD(α) represents the polynomial-based distribution function; PM(α) denotes the polynomial-based variation function; $SE(\alpha^{\prime })$ represents the selection function on the basis of non-dominated sorting. 

### 4.5. The Complexity Analysis of the Local Repair Algorithm

During the local repair process, when the number of new populations exceeds the upper limit of the population, each individual needs to be compared. So it takes a lot of time. When the population is *NP*, the maximum allowable quantity of the offspring is $W_{\max}$. Then, after the population is merged in step 5, the population increases to $NP*W_{\max}$. Based on the selection strategy of non-dominated sorting, the number of comparisons between individuals is ${\left(NP*W_{\max}\right)}^{2}$. In summary, the upper limit of the complexity of the local repair algorithm is $O[{\left(NP*W_{\max}\right)}^{2}]$.

## 5. Global Event Coverage Hole Repair Algorithm

### 5.1. Main Idea

The differential evolution (DE) algorithm is a population-based global search algorithm. The DE algorithm identifies the optimization orientation via the distance and orientation of individuals in the population. The differential evolution algorithm is mainly implemented by the following steps:

1. Population initialization: Randomly and uniformly initialize individuals in a population in the search space.
2. Mutation operation: DE algorithm implements individual variation through differential strategy, which is also an important indicator different from genetic algorithm.
3. Crossover: Introducing crossover operators can enhance population diversity.
  (10) $$e_{i,j}=\left\{ \begin{array}{l} w_{i,j}\;if\;rand(0,1)\leq P_{co}\;or\;j=j_{rand}\\ \alpha_{i,j}\;else \end{array}\right.$$
  $P_{co}$ is the cross probability, $P_{co}\in [0,1]$. $j_{rand}$ is a random integer, $j_{rand}\in [1,2,\ldots ,n]$.
4. Select Operator: In the differential evolution algorithm, the properties of the selection operator are as follows:

Property 1: For each individual, $\alpha_{i}(g+1)$ must be better than $\alpha_{i}(g)$;

Property 2: The algorithm will eventually converge to an optimum (possibly local optimum);

Property 3: Mutation and crossover operations help to jump out of local optimum to reach global optimum.

The differential evolution (DE) algorithm identifies the optimization orientation via the distance and orientation of individuals in the population. So, the distance between individuals is crucial for the variation in DE algorithm. In the optimization space, the location of the individual provides the information about the current optimization capabilities of the algorithm. When there is a long distance between individuals, the distance information between individuals can provide more optimization space; when the distance between individuals is very close, the distance information between individuals can provide a better solution for the local space. Therefore, in the differential evolution algorithm, the weighted distance difference between individuals has the ability to optimize and provide the direction of the optimization. Meanwhile, the cross process makes sure that helpful vector information generated in the variation process is able to be retained. In [35], it has been proved that the variation mechanism "*DE/rand/1*" is always able to evolve to the feasible domain interval and the optimal solution direction. The "*DE/rand/1*" mechanism can be expressed as:

(11) $$w_{k}=\alpha_{s_{1}}+Z(\alpha_{s_{2}}-\alpha_{s_{3}})$$

In Equation (11), $\left\{s_{1},s_{2},s_{3}\}\in rand[1,n\right]$, Z is the regulatory factor. $w_{k}$ is the variation vector generated based on the "*DE/rand/1*" mechanism.

### 5.2. The Global Repair Strategy

When using differential evolution algorithm to deal with multi-constraint network healing issue, it is essential to consider not only feasible solutions but also infeasible solutions. During the optimization procedure, superior infeasible solutions is also able to direct the optimization orientation of the algorithm, for instance the infeasible solutions near the periphery. Therefore, the selection mechanism of the differential evolution algorithm in the global optimization process is defined as:

**Definition 3.**  *When the generated test vector e_k_ is a feasible solution, the test vector e_k_ will compare the objective function value with all feasible solutions in the population. When the objective function value of e_k_ is better than all feasible solutions, the individual α_k_ with the smallest objective function value among all feasible solutions is replaced by e_k_*.

**Definition 4.**  *When the generated test vector e_k_ is an infeasible solution, then the test vector e_k_ will be compared to all infeasible solutions in the population. According to Pareto dominance, when there is no infeasible solution to dominate e_k_, then all non-dominated solutions will be sorted according to the non-dominated sorting algorithm* [36]*. After sorting, the last individual α_k_ is compared to e_k_ again. When the restraint violation degree of α_k_ is greater than e_k_>, then replace α_k_ with e_k_*.

The global repair strategy takes into account not only the feasible solutions but also the infeasible solutions when solving the optimization issue. During the optimization procedure, superior infeasible solutions is also able to provide guidance for the optimization orientation of the algorithm, for instance the infeasible solution close to the periphery. Though two non-dominated infeasible solutions are unable to be compared during the global optimization procedure when settling the restricted optimization issue, the algorithm is still inclined to select the infeasible solution with low restraint violation degree.

The global repair algorithm on the basis of DE algorithm is denoted in Algorithm 2.

**Algorithm 2** The Global Event Coverage Hole Repair Algorithm Based on DE Algorithm1: Begin2:  Input: $\alpha =(\alpha_{1},\alpha_{2},\ldots ,\alpha_{n})\in T\subset R^{n}$;3:  *For k* = 1: *n* do;4:    Select $s_{1},s_{2},s_{3}\in ([1,n]-k)$ randomly; 5:    $w_{k}=Mt(\alpha_{s_{1}},\alpha_{s_{2}},\alpha_{s_{3}},Z)$; 6:    $e_{k}=Co\;(w_{k},\alpha_{k},P_{co})$;7:    Select $\left(e_{k},\alpha_{k}\right)$;8:  End *for*.9: End

In Algorithm 2, $w_{k}$ denotes the variation vector generated by "*DE/rand/1*" mechanism; Z represents the regulatory factor; $e_{k}$ denotes the test vector generated by binomial crossover operator; $P_{co}\in [0,1]$ represents the crossover probability; Mt (α) denotes the variation operator; Co  (α) represents the crossover operator.

### 5.3. The Complexity Analysis of the Global Event Coverage Hole Repair Algorithm

During the global repair process, time is mainly consumed in the loop operation. Set the population *n* = *NP*. When the population are all feasible solutions, according to the repair mechanism based on global optimization, individuals need to compare *NP* times. When the population are all infeasible solutions, according to the repair mechanism based on global optimization, the infeasible solutions need to be sorted again. These infeasible solutions need to be compared *NP*^2^ times in each sorting process. The above infeasible solutions also need to be compared *NP* times after sorting. Therefore, the upper limit of the complexity of the global repair algorithm is $O(NP^{2}+NP)=O(NP^{2})$.

## 6. Joint Event Coverage Hole Repair Algorithm

### 6.1. The Novelty of The Joint Event Coverage Hole Repair Algorithm

- The multi-restraint network healing problem is highly intricate. Therefore, the multi-restraint network healing issue is translated into an unrestricted multiple target healing issue. By transformation, the unrestricted multiple target healing issue is able to be settled by effective multi-objective optimization algorithm, which is able to boost the efficiency of the solutions and simplify the complexity of the issue.
- The proposed method effectively balances the relationship between the objective function and the restraint violation degree by deeming the constraint violation degree as another objective function.
- The joint event coverage hole repair algorithm (JECHR) combines local repair and global repair to heal event coverage holes effectively. In the JECHR algorithm, the IWO algorithm is used for local optimization to make sure the astringency of the optimization procedure. Diversity is a significant index of the population. The DE algorithm is used for global repair to make sure the diversity of repair strategies. The IWO algorithm enables each individual to explore the circumambient information deeply and provide the information to the DE algorithm, facilitating the DE algorithm to accomplish a broader global optimization. The DE algorithm is able to take full advantage of the information shared by the IWO algorithm to seek individuals with superior objective function values. The superior individuals which are sought are provided in the IWO algorithm for more detailed local optimization.

### 6.2. Joint Event Coverage Hole Repair Algorithm

In Algorithm 3, the local healing procedure is conducted before the global healing. So, individuals that have been locally optimized are able to share the information needed for global optimization.

The procedure of the JECHR algorithm is denoted in Algorithm 3.

**Algorithm 3** The Joint Event Coverage Hole Repair Algorithm1: Begin2:  Initialize j=1;3:  Input $\alpha_{1}\in T\subset R^{n}$;4:   $F(\alpha_{1})=(f(\alpha_{1}),V(\alpha_{1}))\;\;$; 5:   *While* repair quality is not satisfied, do; 6:     $\alpha_{j}=LR\;\;(\alpha_{j},F_{j})$;7:     $F(\alpha_{j})=(f(\alpha_{j}),V(\alpha_{j}))$;8:     $\left[\alpha_{j},F_{j}]=GR\;(\alpha_{j},F_{j}\right)$;9:     j=j+1;10:   End *while*.11: End

In Algorithm 3, F(α) denotes the objective function value after transformation; LR (α) represents the local repair algorithm; GR (α) represents the global repair algorithm.

## 7. Performance Evaluation

In the simulation experiment, the performance of the JECHR algorithm is validated by healing event coverage holes in multi-constraint networks. Five mobile robots take five different kinds of sensors setting out from the base point to heal the networks in an optimal route. According to various restraints in four cases, the amount of sensors that are carried by each mobile robot in each case is also different.

### 7.1. Environment Settings

In the simulation experiments, five kinds of sensors are applied. These five different kinds of sensors are smoke sensors, camera sensors, laser sensors, RFID sensors, and infrared sensors. They have various repair time, repair cost, repair energy and repair error depending on their own attributes. Each mobile robot carries one kind of sensor. The parameters of various sensors are listed in Table 1.

In order to compare the performance of the event coverage hole repair algorithm in multi-constrained environment, the parameters of different sensors are decided by the properties of the sensors. There is no related work in event coverage hole repair under multiple restraints. In practice, applying smoke sensors to repair event coverage holes is the fastest. Therefore, the repair time of the smoke sensor is set to 1, which is the smallest of all sensor repair time. In practical applications, it is most accurate to use the camera sensor to repair the event coverage holes. Therefore, the repair error of the camera sensor is set to 5, which is the smallest of all sensor errors. The energy consumption of the RFID sensor is very low. Therefore, the repair energy of the RFID sensor is set to 10, which is the smallest of all sensor repair energy. The infrared sensors are susceptible to unrelated heat sources, resulting in low accuracy. Therefore, the repair error of the RFID sensor is set to 35, which is the largest of all sensor repair errors. In practical applications, there will be many types of smoke sensors, camera sensors, laser sensors, RFID sensors, and infrared sensors, whose parameters may not be the same as the sensor parameters set in the experiment. It can be seen from the performance comparison of the algorithms that the performance of algorithms do not depend on the sensor parameter setting.

### 7.2. Experimental Evaluation

During the healing process, the event coverage hole repair algorithms not only need to satisfy the restraints of repair time, repair cost, repair energy, and repair error, but also need to heal event coverage holes in the optimal path. Next, the influence of the number of mobile robots on event coverage hole repair is tested. In the performance comparison experiment, the JECHR algorithm is compared with the MOACO [37], iMOGA [38], and SOS-SA [39] algorithms in the same experimental environment. The relationship between the repair path distances under different number of event coverage holes, repair time restraints and repair error restraints is compared.

The simulation experiments on event coverage hole repair are carried out in four different experimental cases to test the repair performance under different restraint conditions. Each case is subject to different repair restraints. The experimental parameters are presented in Table 2. The different experiment conditions are set to evaluate the performance of the proposed algorithm in a variety of multi-constraint environments, which are subject to diverse repair time, repair cost, repair energy, and repair error restraints. The experiment conditions are set according to the parameters of sensors in Table 1. The experiment conditions are set to test whether the proposed algorithm can reasonably allocate sensor resources in multi-constraint environments. In practical applications, the event coverage hole repair process will face a variety of multi-constraint environments. The experiment conditions in the manuscript are part of multi-constraint environments in practical applications.

**Figure 1:**
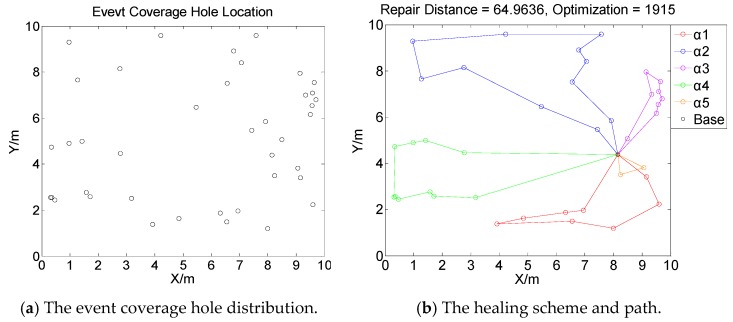
Case 1: The network repair performance and path applying mobile robots and diverse kinds of sensors in multi-constraint environment.

**Figure 2:**
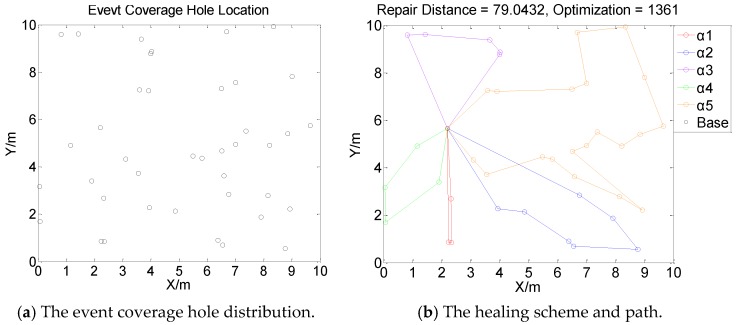
Case 2: The network repair performance and path applying mobile robots and diverse kinds of sensors in multi-constraint environment.

**Figure 3:**
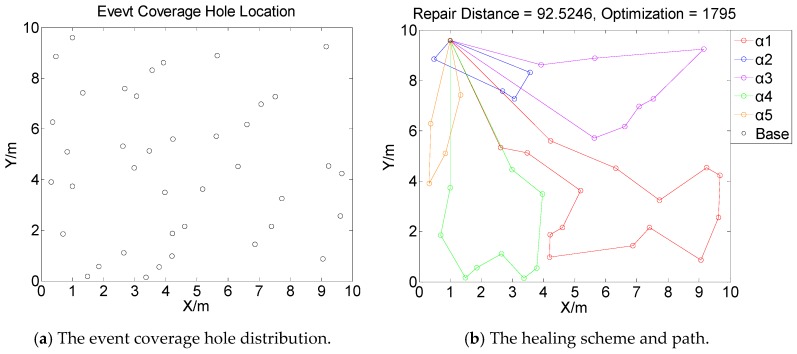
Case 3: The network repair performance and path applying mobile robots and diverse kinds of sensors in multi-constraint environment.

**Figure 4:**
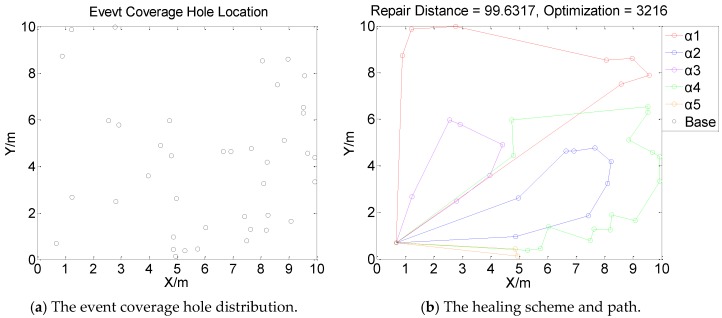
Case 4: The network repair performance and path applying mobile robots and diverse kinds of sensors in multi-constraint environment.

Figure 1, Figure 2, Figure 3 and Figure 4 perform the effect and path of network healing in four multi-constraint scenarios. Figure 1a, Figure 2a, Figure 3a, and Figure 4a demonstrate four different event coverage hole distribution in the networks. The circles in the figure represent the position of the event coverage holes. Figure 1b, Figure 2b, Figure 3b and Figure 4b demonstrate the healing scheme and path for four multi-constraint cases. Five mobile robots that take five type of sensors set out from the base point synchronously. Each mobile robot takes one kind of sensor to heal forty event coverage holes in the network. The various colored paths in Figure 1b, Figure 2b, Figure 3b and Figure 4b denote the healing path of five mobile robots. 

In practical applications, event coverage holes will occur after monitoring tasks or monitoring demands change. Event coverage holes can occur anywhere in the surveillance area based on different monitoring tasks and demands. The location of the event coverage holes is random. Therefore, the location of the event coverage holes is set to appear randomly to test whether the proposed algorithm can meet the practical application demands. The event coverage hole repair algorithm needs to accommodate the event coverage holes that may occur anywhere in the surveillance area. In the experiments, the position coordinates of the forty event coverage holes are randomly generated in the surveillance area to test whether the proposed algorithm can repair the event coverage holes under any location distribution.

In [40], the authors consider the problem of planning paths for unicycle robots with dynamic model. In practical applications, it is not possible for all the robots to turn into any headings. In the experiment, we simplify the robots to be points, which are only suitable for robots that can move in all directions. When healing event coverage holes in four cases, it is required to satisfy multiple repair restraints synchronously. In Figure 1, the first case for the repair error restraints is the most rigorous in four cases. Besides, the healing procedure of the networks is not merely subject to rigorous error restraints, but also restricted by repair time, cost, and energy restraints. Thus, the camera sensors, which have less repair error during the healing process of networks, are more used, with a total of 11; in Figure 2, the second case for repair cost restraints is the severest of the four cases. The healing process of the event coverage holes is not only subject to severe error restraints, but also restricted by the other three repair restraints. Thus, the infrared sensors that have lower repair cost are more applied, with a total of 20; in Figure 3, the third case for the repair time restraints are the most rigorous of the four cases during the network healing procedure. At the same time, repair cost, energy, and error restraints also need to be satisfied. Thus, the smoke sensors, that have less repair time, are most applied, with a total of 15; in Figure 4, the repair energy restraints are the most rigorous of the above four cases. It must also satisfy the other three restraints.Therefore, the RFID sensors with less energy consumption are most applied. 

During the healing procedure, the global repair algorithm on the basis of DE algorithm is applied to make sure the diversity of the healing mechanism. The local repair algorithm on the basis of the IWO algorithm ensures the astringency of the healing process. Thus, the JECHR algorithm on the basis of DE algorithm and IWO algorithm is able to heal the networks effectively in multi-constraint environment.

**Figure 5:**
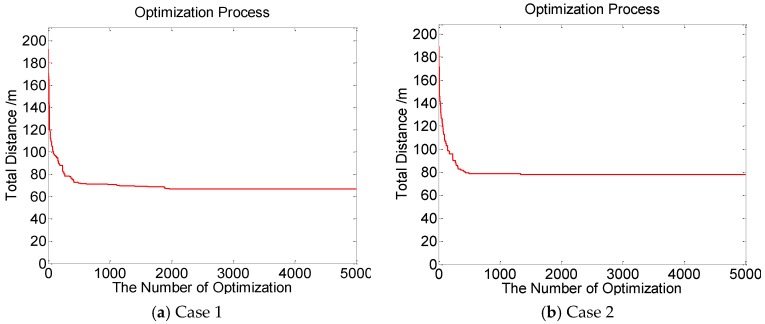

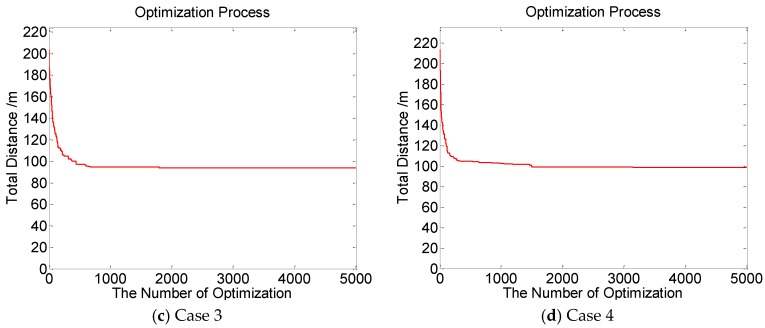
Four cases of the optimization process of the event coverage hole repair route by mobile robots in multi-constraint environment.

The relationship between the repair path distance by mobile robots and the optimization process in the four multi-restraint cases is shown in Figure 5. It indicates that in the initial stage of the optimization process, the repair route distance of the event coverage holes decreases rapidly. As the repair process continues, the distance of the repair route decreases slowly and tends to be stable finally. In the optimization process, the repair distance of event coverage holes declines steadily. There is no problem of trapping into local optimum and continuous oscillation. The global repair algorithm on the basis of DE algorithm is able to optimize the global repair route quickly in the initial stage. At the same time, the local repair algorithm on the basis of IWO algorithm is able to prevent the optimization process from trapping into local optimum and make sure the astringency of the optimization process. In Figure 5a, the first case is optimized after 1915 times. The repair distance of the event coverage holes is 64.9636 m; in Figure 5b, the second case is optimized after 1361 times. The repair distance of the event coverage holes is 79.0432 m; in Figure 5c, after 1795 optimization processes, the repair distance of the third case is 92.5246 m; in Figure 5d, the fourth case passes through 3216 optimization processes. The repair distance is 99.6317 m.

**Figure 6:**
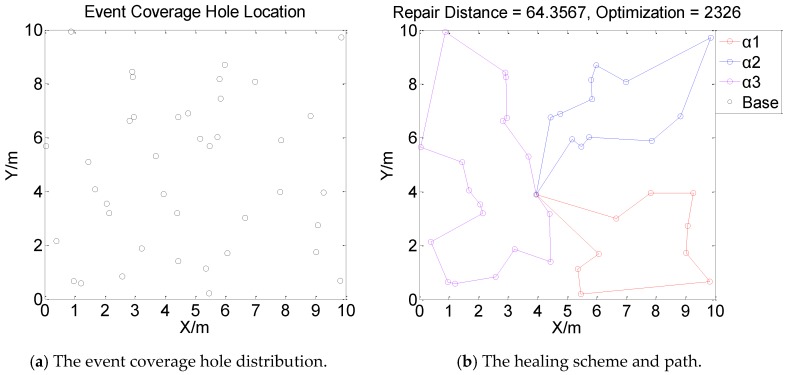
The event coverage hole repair performance and route using three mobile robots.

**Figure 7:**
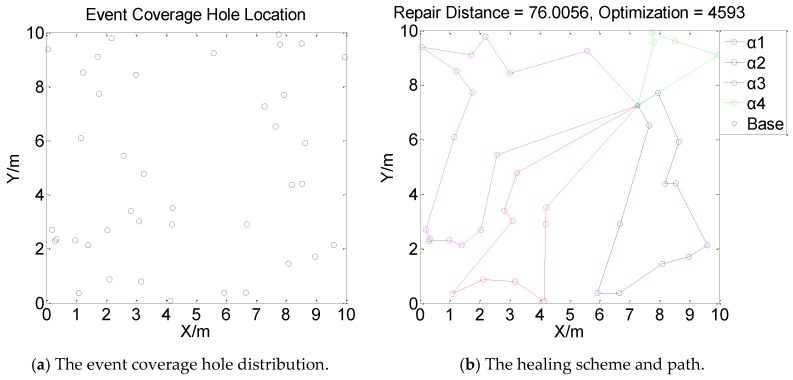
The event coverage hole repair performance and route using four mobile robots.

**Figure 8:**
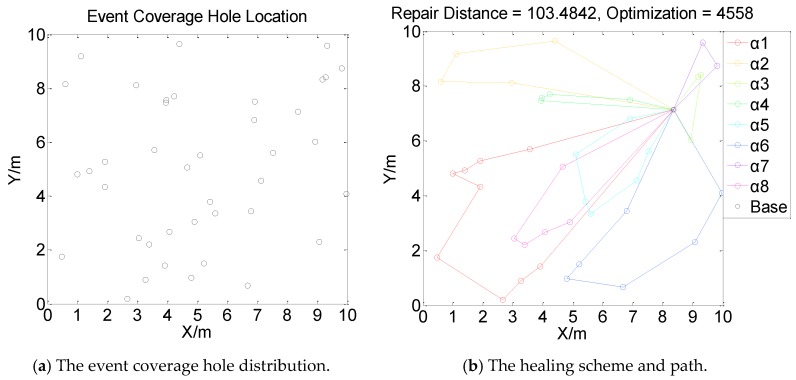
The event coverage hole repair performance and route using eight mobile robots.

Next, in order to verify the effect of the amount of mobile robots on the event coverage hole repair performance, three, four, and eight mobile robots are applied to heal forty event coverage holes respectively, as shown in Figure 6, Figure 7 and Figure 8.

Figure 6, Figure 7 and Figure 8 indicate that the repair path distance gradually increases as the number of mobile robots in the networks increases. While, the optimization times are also rising. This is due to all mobile robots need to set out from the base point. Compared with moving from repaired event coverage holes to the unrepaired event coverage holes, setting out from the base point and returning to the base point add extra distance which leads to an increase in repair path distance. During the process of healing networks, mobile robots are subject to multiple restraints. The repair strategy need to weigh the relationship between multiple restraints and repair paths. Therefore, the distance of the repair path by each mobile robot is not relatively balanced. In Figure 6, Figure 7 and Figure 8, according to different distribution of event coverage holes in the network, the mobile robots select the optimal starting point as the base point on the basis of the total repair path distance.

As the number of mobile robots in the network increases, the complexity of path planning increases rapidly, which eventually leads to an increase in optimization times. In practical applications, different kinds of sensors are needed to heal the network. On the basis of the characteristic and number of sensors, multiple mobile robots are needed to heal event coverage holes in the network. Although the total repair distance of mobile robots increases, the repair performance and repair speed of multiple mobile robots will be greatly improved, especially for the application scenarios that have strict demands for real-time repair.

### 7.3. Comparison with the MOACO, iMOGA and SOS-SA Algorithms

In this section, for purpose of verifying the performance of the JECHR algorithm, the proposed algorithm is compared with the MOACO algorithm and the latest iMOGA, SOS-SA algorithms under different number of event coverage holes [37,38,39,41]. The representation of the candidate solutions for each method (JECHR, MOACO, iMOGA, SOS-SA) is listed in Table 3, Table 4, Table 5 and Table 6. The parameters used in each method under comparative study are listed in the in Table 7, Table 8, Table 9 and Table 10.

**Figure 9:**
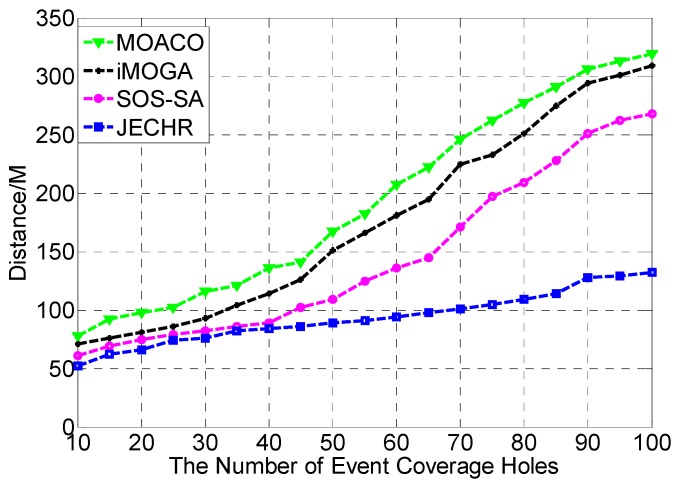
The JECHR algorithm is compared with MOACO, iMOGA, and SOS-SA algorithms under different number of event coverage holes.

As Figure 9 shows, in the initial stage, as the number of event coverage holes continues to increase, the repair path distances of the four algorithms increase rapidly. When the number of event coverage holes in the network exceeds 90, the repair path distance growth of the SOS-SA algorithm and the JECHR algorithm tends to be flat. This is due to when the amount of event coverage holes in the network is small, the density of event coverage holes is low. Mobile robots need to move longer distances to heal event coverage holes in the network. As the amount of event coverage holes increases, the density of event coverage holes in the network increases gradually. Finally, the network tends to be saturated. The SOS-SA algorithm and JECHR algorithm continuously optimize the repair path, which gradually slows down the growth of the repair path distance. The repair effect of the JECHR algorithm is obviously superior to the MOACO, iMOGA, and SOS-SA algorithms. The performance of the MOACO algorithm is the worst. In the changing network environment, the parameters α and β of the MOACO algorithm cannot be adaptively adjusted according to different network environment. Therefore, the solving speed is slow. The quality of solutions are poor. The SOS-SA algorithm is based on simulated annealing algorithm, which is a greedy algorithm essentially. Therefore, its parameters are difficult to control. There is no guarantee that it can converge to the optimum solution once. The SOS-SA algorithm usually takes multiple attempts to get the optimum solution that is likely to trap into local optimum. The iMOGA algorithm is a multi-objective genetic algorithm, which has a certain dependence on the initial population selection. In the mean time, the multi-objective genetic algorithm is prone to premature phenomenon. The algorithm has limited search ability for new space and is easy to converge to local optimum. Therefore, when there are plenty of event coverage holes in the network, it is impossible for iMOGA algorithm to provide an optimal repair solution for the network.

To verify the performance of the JECHR algorithm in the face of different restraints, the JECHR algorithm is compared with the MOACO algorithm and the latest iMOGA, SOS-SA algorithms under different single repair time and repair error restraints. Currently, there is no related work in event coverage hole repair under multiple restraints. 

**Figure 10:**
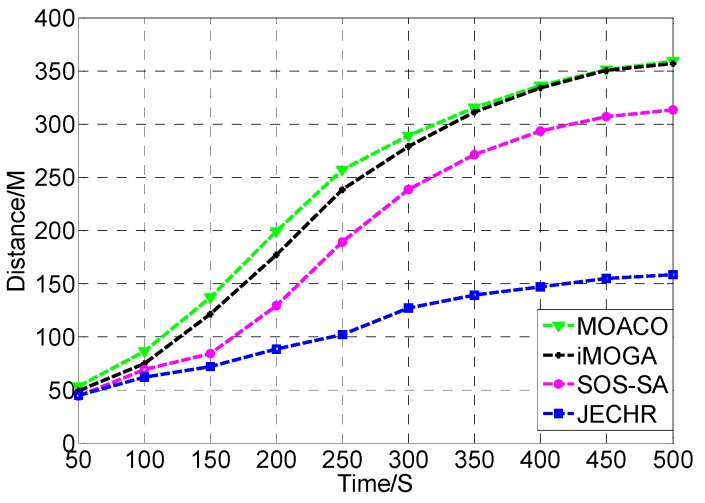
The JECHR algorithm is compared with MOACO, iMOGA, and SOS-SA algorithms under different repair time restraints.

As Figure 10 shows, the repair time restraint continues to increase. More and more sensors need to be deployed to heal the event coverage holes in the network. Mobile robots need to move longer distances to repair the network. The repair effect of the JECHR algorithm is superior to the MOACO, iMOGA, and SOS-SA algorithms. The MOACO algorithm converges slowly and is likely to trap into local optimum. In the mean time, the ant colony algorithm needs a long optimization time. In the optimization process, the MOACO algorithm is prone to stagnation, which cannot find a better solution. The performance of the SOS-SA algorithm is greatly influenced by the repair time restraint. The SOS-SA algorithm is based on simulated annealing algorithm which is also a greedy algorithm essentially. The simulated annealing algorithm has poor global optimization ability, which is susceptible to parameters. The iMOGA algorithm is easy to premature, which has limited search ability for new space. It is prone to converge to local optimum. Therefore, when there are many event coverage holes that need to be repaired in the network, the iMOGA algorithm is likely to trap into local optimal solution. The repair path cannot be further optimized. The JECHR algorithm applies the DE algorithm for global repair to make sure the diversity of the healing strategy. Meanwhile, the IWO algorithm is used for local optimization to make sure the astringency of the optimization procedure. Therefore, the JECHR algorithm can heal the event coverage holes with the shortest repair path distance under different repair time restraints. 

**Figure 11:**
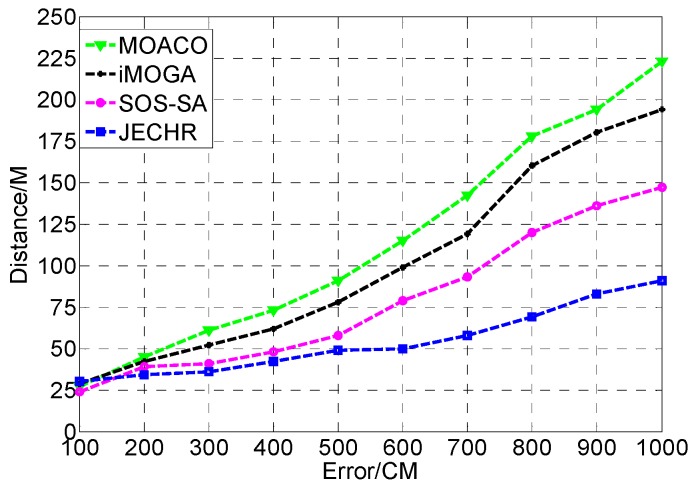
The JECHR algorithm is compared with MOACO, iMOGA and SOS-SA algorithms under different repair error restraints.

As Figure 11 shows, as the error restraints increase, more and more sensors need to be deployed to heal the event coverage holes in the networks. Then, mobile robots need to move longer distances to complete the network repair. The repair path distance of the JECHR algorithm is obviously superior to MOACO, iMOGA, and SOS-SA algorithms. The MOACO algorithm converges slowly and is likely to trap into local optimum. In the optimization process, the MOACO algorithm is prone to stagnation which cannot find a better solution. The iMOGA and SOS-SA algorithms are affected by the error restraint greatly. The iMOGA algorithm has a certain dependence on the initial population selection. In the meantime, the multi-objective genetic algorithm is prone to premature and easily converges to the local optimal solution. The global optimization ability of the SOS-SA algorithm is poor. Meanwhile, it is susceptible to parameters. The JECHR algorithm can be adapted to different repair error restraints. Moreover, conventional penalty function technique cannot effectively weigh the size of the penalty factor. The JECHR algorithm can effectively overcome the problem that the penalty factor has function dependence and poor generality. Therefore, the JECHR algorithm can effectively overcome the impact of increased network repair error on the repair path distance. 

## 8. Conclusions

In this paper, a joint event coverage hole repair algorithm on the basis of global repair and local repair is presented to apply mobile robots to heal event coverage holes in WSRNs. The differential evolution algorithm is applied for global repair to make sure the diversity of the healing mechanism. The invasive weed optimization algorithm is used for local repair to make sure the astringency of the optimization procedure. The JECHR algorithm efficiently solves the healing issue of the event coverage holes by applying mobile robots in multi-constraint environment. In the end, the simulation experiments demonstrate that the proposed JECHR algorithm is able to heal the event coverage holes effectively in intricate multi-constraint environment.

## Figures and Tables

**Table 1:** The parameters of sensors.

Symbol	Meaning	T/s	C	En/J	Er/CM
*α*_1_	Smoke Sensor	1	20	20	20
*α*_2_	Camera Sensor	4	8	15	5
*α*_3_	Laser Sensor	3	12	25	15
*α*_4_	RFID Sensor	2	16	10	10
*α*_5_	Infrared Sensor	5	4	30	35

In Table 1, the abbreviation denotes as follows: T: repair time (T/s); C: repair cost (C); En: repair energy (En/J); Er: repair error (Er/CM).

**Table 2:** The parameters of the experiments.

Case	T/s	C	En/J	Er/CM	Dis/M
1	106	512	685	505	64.9636
2	154	320	930	910	79.0432
3	90	576	745	655	92.5246
4	99	540	630	530	99.6317

In Table 2, the abbreviation denotes as follows: T: repair time restraints (T/s); C: repair cost restraints (C); En: repair energy restraints (En/J); Er: repair error restraints (Er/CM); Dis: total repair distance (Dis/M).

**Table 3:** The representation of the candidate solutions in terms of the distance and the number of event coverage holes.

Algorithm/Number	10	15	20	25	30	35	40	45	50	55
JECHR	52	62	66	74	76	82	84	86	89	91
MOACO	78	92	98	102	116	121	136	141	167	182
iMOGA	71	76	81	86	93	104	114	126	151	166
SOS-SA	61	69	75	79	82	86	89	102	109	125

**Table 4:** The representation of the candidate solutions in terms of the distance and the number of event coverage holes.

Algorithm/Number	60	65	70	75	80	85	90	95	100
JECHR	94	98	101	105	109	114	128	129	132
MOACO	207	222	246	262	277	291	306	313	319
iMOGA	181	195	225	233	251	275	294	301	309
SOS-SA	136	145	171	197	209	228	251	262	268

**Table 5:** The representation of the candidate solutions in terms of the distance and the repair time restraints.

Algorithm/Repair Time Restraints	50	100	150	200	250	300	350	400	450	500
JECHR	45	62	72	88	102	127	139	147	155	158
MOACO	53	86	137	199	257	289	315	336	351	359
iMOGA	49	75	121	177	238	279	311	334	350	357
SOS-SA	44	69	84	129	189	238	271	293	307	313

**Table 6:** The representation of the candidate solutions in terms of the distance and the repair error restraints.

Algorithm/Repair Error Restraints	100	200	300	400	500	600	700	800	900	1000
JECHR	30	34	36	42	49	50	58	69	83	91
MOACO	27	45	61	73	91	115	142	178	194	223
iMOGA	29	42	52	62	78	99	119	160	180	199
SOS-SA	24	39	41	48	58	79	93	120	136	154

**Table 7:** The parameters of the JECHR algorithm in the comparative experiments.

Symbol	Meaning	Parameters
*W*_max_	Maximum Number of Offspring	3
*W*_min_	Minimum Number of Offspring	0
*P*_int_	Initial Population	30
*P*_max_	Maximum Population	80
*Z*	Regulatory Factor	0.8
*P_co_*	Crossover Probability	[0.8, 1]
*PD*	Polynomial Distribution index	100
*PV*	Polynomial Variation index	1
*P_m_*	Mutation Probability	1/*n*

In Table 7, *n* represents the decision vector dimension.

**Table 8:** The parameters of the MOACO algorithm in the comparative experiments.

Symbol	Meaning	Parameters
*α*	Relative Importance of Pheromone Trail	1
*β*	Relative Importance of Heuristic Information	2
*ρ*	Pheromone Evaporation Rate	0.2
*q*_0_	Relative Importance of Exploration versus Exploitation	0.98
*n*	Number of Ants	10
*m*	Number of Iterations	100
*η*	Heuristic Information	$1/d_{ij}$

In Table 8, $d_{ij}$ represents the distance between the city *i* and *j*.

**Table 9:** The parameters of the iMOGA algorithm in the comparative experiments.

Symbol	Meaning	Parameters
*α*	Left Spreads of LR-fuzzy Variables	0.95
*β*	Right Spreads of LR-fuzzy Variables	0.95
$P_{\max}$	Maximum Generation	1000
$G_{\max}$	Max-Popsize	100
γ	Permissible Probability Levels	0.9
*K*1	Weights of Mean	0.5
*K*2	Weights of Variance	0.5

**Table 10:** The parameters of the SOS-SA algorithm in the comparative experiments.

Symbol	Meaning	Parameters
*K*	Benefit Factor	1
*P*	Population Size	50
$m_{\max}$	Maximum Iteration	1000
$T_{\mathrm{int}}$	Initial Temperature	0.025
*C_r_*	Cooling Rate	0.99
